# Hydrogel for the Controlled Delivery of Bioactive Components from Extracts of *Eupatorium glutinosum* Lam. Leaves

**DOI:** 10.3390/molecules28041591

**Published:** 2023-02-07

**Authors:** Lizbeth Zamora-Mendoza, Santiago Nelson Vispo, Lola De Lima, José R. Mora, António Machado, Frank Alexis

**Affiliations:** 1 School of Biological Sciences & Engineering, Yachay Tech University, Urcuquí 100119, Ecuador; 2 School of Chemical Sciences and Engineering, Yachay Tech University, Urcuquí 100119, Ecuador; 3 Departamento de Ingeniería Química, Colegio de Ciencias e Ingenierías, Universidad San Francisco de Quito (USFQ), Quito 170901, Ecuador; 4 Laboratorio de Bacteriología, Instituto de Microbiología, Colegio de Ciencias Biológicas y Ambientales (COCIBA), Universidad San Francisco de Quito (USFQ), Quito 170901, Ecuador

**Keywords:** controlled delivery system, antioxidant activity, anti-hemolytic activity, antibacterial activity, secondary metabolites

## Abstract

This research reported a hydrogel loaded with the ethanolic and methanolic extracts of *Eupatorium glutinosum* Lam. The *E. glutinosum* extracts were characterized by phytochemical screening, Fourier-transform infrared spectroscopy (FTIR), thin-layer chromatography (TLC), and UV/Vis profile identification. This research also evaluated the pharmacological activity of the extracts using antimicrobial, antioxidant, and anti-inflammatory assays prior to polymeric encapsulation. Results indicate that extracts inhibit the *Escherichia colii* DH5-α (Gram negative) growth; excellent antioxidant activity was evaluated by the ferric reducing power and total antioxidant activity assays, and extracts showed an anti-hemolytic effect. Moreover, the cotton and microcrystalline cellulose hydrogels demonstrate successful encapsulation based on characterization and kinetics studies such as FTIR, extract release, and swelling degree. Moreover, effective antibacterial activity was registered by the loaded hydrogel. The overall results encourage and show that *Eupatorium glutinosum*-loaded hydrogel may find a wide range of bandage and wound healing applications in the biomedical area.

## 1. Introduction

Since ancient times, plants have been used by the population for medical and health purposes. Ecuador is recognized as a megadiverse world country because of its highest biodiversity [1]. Ecuadorian medicinal plants—located in South America—have been used for anti-hemolytic, antibacterial, and antioxidant applications to treat diseases as traditional medicine [2]. For this reason, plant-derived metabolites have been studied for their potential application as a source of new drug products due to the large variety of species and the identification of unknown metabolites secondary to anti-hemolytic, antioxidant, and antibacterial therapeutic properties [3].

There are two types of extracts: crude plant extracts and single compound isolation. Both extracts are characterized by drug discovery in the pharmaceutical industry. Sometimes, it is challenging to isolate active constituents from plant extracts due to inadequate fraction processing as well as the degradation of the biocomponents that modify the biological activity [4]. Therefore, the whole extract is used to evaluate therapeutic effects that could also suggest the synergetic interaction or multi-factorial mixture of biomolecules in herbal extracts [5]. Then, the molecular mechanism of the bioactivity of plant extracts is essential for their clinical application, efficacy, and commercialization. The ethnobotanical effects conferred by human history are linked to the side effects of pure chemicals. Natural plant extracts could have a similar potency as synthetic drugs, but the adverse effects could be achieved by using larger quantities or for longer period of time [6]. A polymeric system for controlled extract release can be used to increase the bioavailability of extracts with anti-hemolytic, antioxidant, and antibacterial activity.

*Eupatorium* genus from Asteraceae family have been used in traditional medicine in many parts of the world [7,8]. The *Eupatorium glutinosum* Lam. is a plant with medicinal application corresponding to anti-inflammatory and antiseptic in the Ecuadorian community [9]. The vernacular name is Matico [10]. This plant grows to approximate altitudes that reach 3000 m above sea level (masl). Reports indicated the use of *E. glutinosum* to cure diarrhea, headaches, and stomach ulcers. Studies related to the *Eupatorium* genus indicate the presence of flavonoids, lactones, terpenoids, alkaloids, and their derivates [11]. This type of secondary metabolite could be responsible for astringent, antirheumatic, and antimicrobial uses [12].

The current challenge is finding the functional encapsulation method for applying extracts in medicine. For this reason, one alternative is using polymeric biomaterials such as hydrogels to increase pharmaceutical applications. Hydrogels are three-dimensional polymeric networks with crosslinked structures that can absorb water [13]. Hydrogels can be obtained from natural or synthetic polymers. Natural polymers such as chitosan, starch, and cellulose have the advantages of biocompatibility, mechanical properties, density, and swelling degree [14].

Thus, this study focuses on enhancing the potential therapeutic use of the medicinal plant-based natural compounds studied through an encapsulation method to achieve easy administration, low production cost, and controlled release.

## 2. Results and Decision

### 2.1. Extract Preparation

The extract percentage yield using methanol as solvent was 8.97%. Meanwhile, the dried plant materials with ethanol yielded 6.37% (see Table 1). Organic solvents (methanol and ethanol) were used based on other studies [15]. Among the evaluated solvents, methanol resulted in the highest extraction yield. It could indicate that the highly polar solvents favor extraction efficiency [16].

### 2.2. Phytochemical Screening

The phytochemical screening of *Eupatorium glutinosum* Lam. alcoholic extracts revealed the presence of several secondary metabolites. Quaternary alkaloids, saponins, terpenoids, and phenols were detected in both samples (see Table 2). These bioactive compounds are known to have a pharmacological interest [17]. Then, it can be mentioned that the solvent influences the content of the secondary metabolites obtained, although the change in their polarities is small. The methanol and ethanol extract tested positive for the majority of phyto-metabolites and its content determined the therapeutic effectiveness of medicinal properties [18].

The EG-methanolic extract had a high concentration of quaternary alkaloids, saponins, terpenoids, and phenols. On the other hand, sample EG-ethanolic had a high content of amino acids as determined by the Dragendorff test, as well as saponins, steroids, terpenoids, quinones, and phenols. Flavonoids, steroids, and terpenoids have anti-inflammatory properties [19]. Terpenoids are related to antimicrobial, antifungal, and anti-allergenic applications [20]. Then, the polarity and solvent used in the extraction process can affect the medicinal properties of *Eupatorium glutinosum* Lam. and their potential therapeutic applications. As reported in a previous study [10], the bioactive compounds characterized in *E. glutinosum* could be beneficial for several biomedical uses.

### 2.3. Thin Layer Chromatography of Extracts

**Figure 1:**
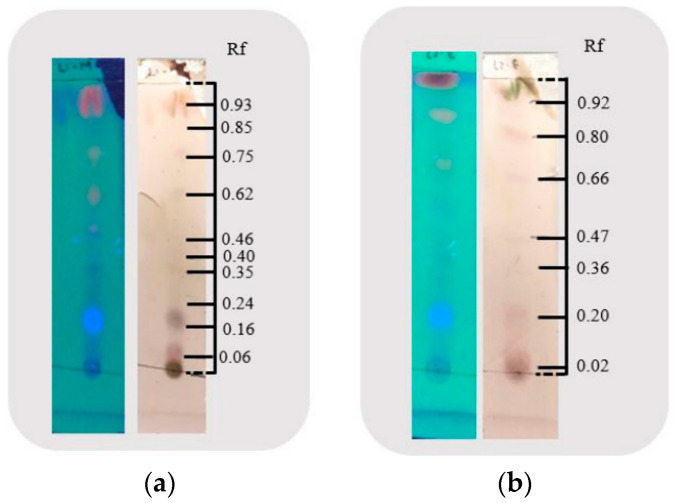
Thin-layer chromatographic of (**a**) EG-methanolic and (**b**) EG-ethanolic extracts.

The thin-layer chromatography (TLC) of *Eupatorium glutinosum* Lam. alcoholic extracts was applied to the silica gel at 60 F_254_ for 30 min to separate extremely non-polar substances and analyze the substances. The Rf corresponding to EG-methanolic and EG-ethanolic demonstrated that the extracted content is influenced by the solvent [21]. As a result, the methanolic extract showed more compound separation based on polarity than ethanolic extracts. It was registered that Rf 0.06 in EG-methanolic and Rf 0.02 in EG-ethanolic correspond to the same compound. Meanwhile, the Rf 0.16 and Rf 0.24 values in EG-Methanolic correspond to the Rf 0.20 compound in EG-Ethanolic when sulfuric acid/vanillin revealing. Moreover, it was identified that a high concentration of compounds was exhibited at Rf 0.16 and Rf 0.20 in EG-methanolic and EG-ethanolic, respectively, when using a UV-light lamp (365 nm). As cited above, the TLC of the methanolic extract reflects a greater mixture of chemical components than the ethanolic extraction [22]. Using the ultraviolet light lamp at 365 nm in EG-methanolic, the outstanding chemical compound of the extracts was obtained at Rf 0.16, while in EG-ethanolic, it was at Rf 0.20. Figure 1 shows the UV-light lamp at 365 nm (blue plate) and the sulfuric acid/vanillin (cream plate). To conclude, the TLC for EG-methanolic and EG-ethanolic provides a rapid separation compound that indicates the nature of the plant extract components, which are subsequently characterized by UV/Vis spectrum and FTIR.

### 2.4. Spectrophotometric Characterization

#### UV/Vis Spectrum of Extracts

**Figure 2:**
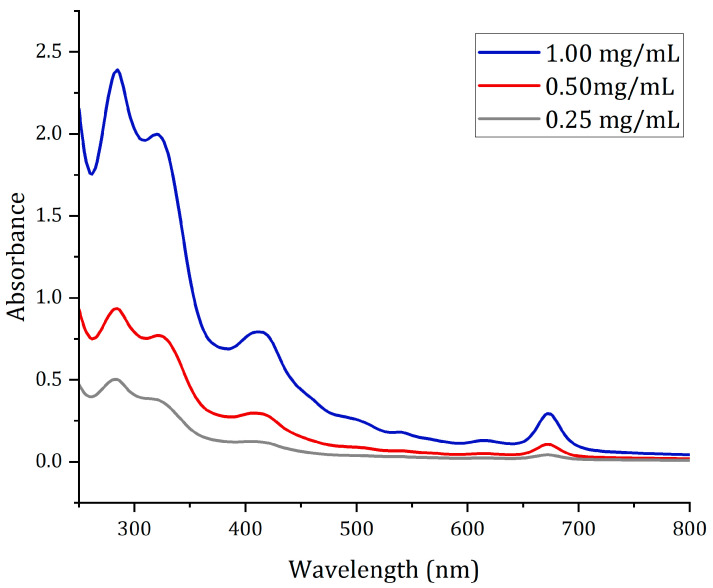
UV/Vis profile of EG-ethanolic sample.

The UV/Vis spectra results showed the presence of phytoconstituents in methanolic and ethanolic extracts in the 250–800 nm range. The raw data were processed with Origin Pro 2021b, the peaks, and the proper baseline with a slit width of 5 nm. UV/Vis spectra analysis corresponds to recognizing peaks in the 200 to 400 nm range (see Figure 2). This region indicates the presence of sulfur (S), nitrogen (N), and oxygen (O). The presence of these elements indicates heteroatoms and unsaturated groups [23]. In fact, the UV/Vis spectrum was used to identify components containing σ bonds, π bonds, lone electron pairs, and aromatic compounds [24]. According to the results of the two plant extracts, it can be deduced that similar UV/Vis profiles were obtained, although the extraction solvent was different since the compounds were similar (see Figure 2). Therefore, the same entity is obtained from the signal.

Therefore, corresponding to the data presented in Table 3, the wavelength number allowed to identify the presence of different secondary metabolites. The UV/Vis spectrum may have identified metabolites corresponding to flavonoids at 320 nm and phenolic compounds at 280 and 360 nm for EG-methanolic and EG-ethanolic extracts (see Table 3). The peak absorbance of chlorophyll at 670 nm corresponds to the green/brown color that characterizes the extracts.

### 2.5. Fourier-Transform Infrared Spectroscopy Spectrum

**Figure 3:**
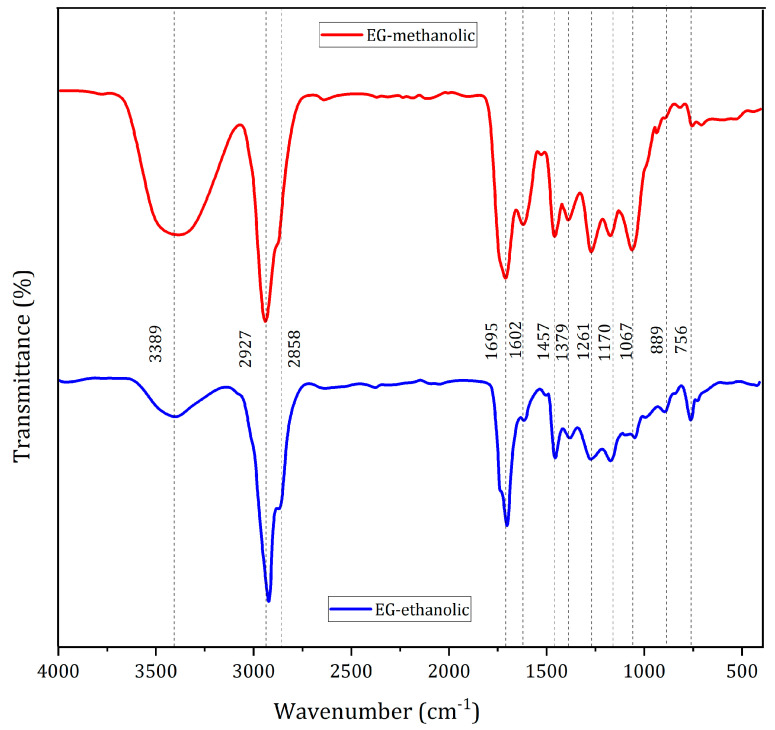
FTIR pattern of *Eupatorium glutinosum* Lam. plant extract.

The Fourier-transform Infrared spectroscopy (FTIR) spectrum of the extracts is helpful in identifying functional groups of the bioactive compounds by analyzing the % transmittance infrared radiation in the region registered. The O-H, -CH, C-O, C=O, C-N, PO3, C-CL, and C=C bonds could correspond to the stretching vibration designated hydroxyl compounds, alcohols, phenols, and aromatic phenols, amino groups, alkanes, or phosphate ions by wavenumber [28]. EG-methanolic and EG-ethanolic extracts are samples from the same plant with different solvents in the extraction step and showed similar peaks from 3600 cm^−1^ to 2000 cm^−1^ (see Figure 3). The recorded result shows distinctive patterns in the fingerprint region (EG-methanolic: C=C–C, phosphate, =C-H, and C-CL) compared to EG-ethanolic (C-O stretch, -CH2X). A clear band for ester groups (1737 cm^−1^) is found for the case of the EG-ethanolic extract (see Figure 3b), while for the case of the methanolic extract, the presence of ketones and aldehydes can be highlighted at 1606 cm^−1^ (see Figure 3a). The broad band near 3300 cm^−1^ is associated with the presence of OH groups from alcohols and carboxylic acids. The bands near 2900 cm^−1^ suggest the presence of C-H from *sp3* and *sp2* carbons [29].

### 2.6. Antibacterial Activity

#### 2.6.1. Agar-Diffusion Essay

The EG-methanolic and EG-ethanolic extracts were effective against *E. coli* DH5-α (see Table 4). The same extracts showed no activity against *E. coli* TG1 and E410A5 strains. EG-methanolic and EG-ethanolic extracts showed antibacterial activity against *E. coli* DH5-α at a 0.25% (*w*/*v*) concentration.

**Figure 4:**
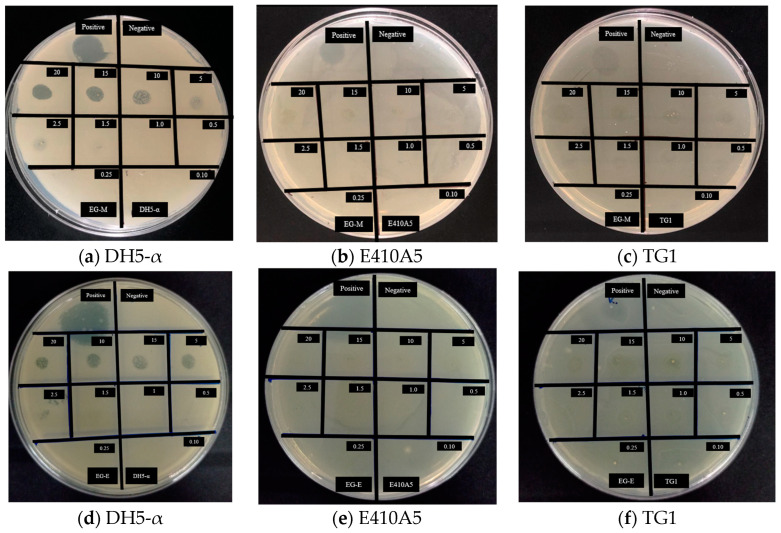
Antibacterial activity on *E. coli* strains by EG-methanolic and EG-ethanolic plant extracts. (**a**–**c**) are related to EG-methanolic extract, and (**d**–**f**) are EG-ethanolic extract.

The different inhibition zone values depend on the concentration of the extract and the chemical and volatile nature of the bioactive constituents (see Figure 4). It suggested that the functional groups identified by FTIR in the methanolic and ethanolic extracts suppressed the *E. coli* DH5-α growth.

The antimicrobial effects of both extracts were compared to those of the positive control. Both extracts showed a significant difference concerning the antibacterial effect of kanamycin. As Gonelimali et al. [30] explained, the difference in the inhibition halo value could correspond to the variation of chemical constituents and the volatile nature of plant extract components. *E. coli* DH5-α have been developed with several mutations to obtain high-efficiency transformation [31]. Many pores and appendices present in the cellular envelope of DH5-α bacteria could confer the distinctive antibacterial response to the extracts from the other strains [32,33].

This method of antibacterial activity is affected by the complex formed by the bioactive components in both extracts. Therefore, inoculum size and concentration may produce a large area of inhibition at lower concentrations, while a thick layer of cells may produce a much smaller inhibition halo or false negatives [34]. As a result, EG-methanolic and EG-ethanolic extracts can be used in biomedical applications related to wound infections.

#### 2.6.2. Time-Kill Curve Analysis

**Figure 5:**
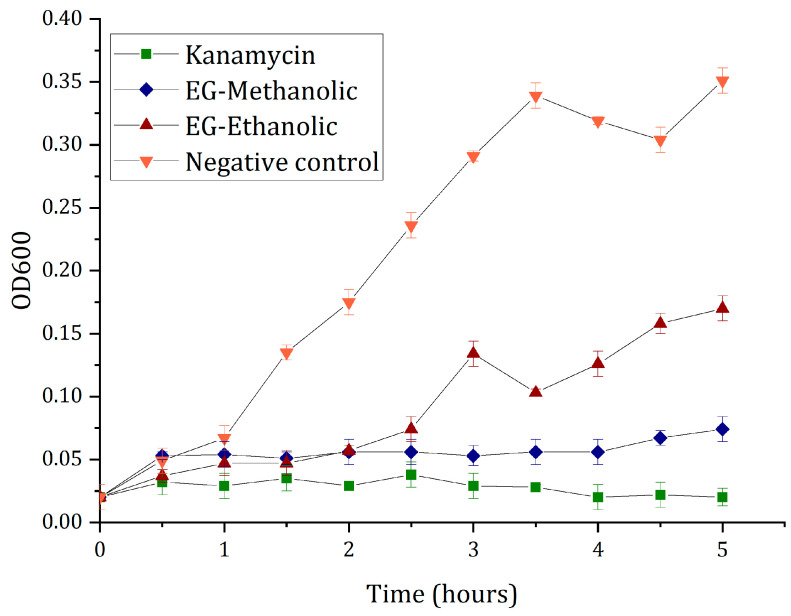
Time-kill curves analysis of the plant extract against *E. coli* DH5-α growth.

After inoculation, bacterial culture tubes (*E. coli* DH5-α growth) were taken to the MaxQ™ 4450 Benchtop Orbital Shaker (Thermo Scientific™, Waltham, MA, USA) at 180 rpm and 37 °C. Once there, an aliquot of 9 µL was used from each cell culture tube every half hour to measure the OD600 value. The results were processed and recorded in Figure 5. The total time of experimental measurements for the time-kill curve analysis was 5 h.

The cell density is directly proportional to the optical density during the initial bacterial growth (i.e., until the absorbance is below 1.0 at 600 nm, also known as OD600). Therefore, as predicted, the negative control OD600 value, where LB broth, Miller was only cultured with DH5-α, showed average bacteria growth without any inhibition signals. In comparison, the positive control showed the expected time-kill curve through the well-known antimicrobial activity of kanamycin. Moreover, EG-methanolic and EG-ethanolic extracts showed antibacterial activity, particularly EG-methanolic extract (see Figure 5). However, the EG-ethanolic extract sample showed low antibacterial activity with values intermediate between the positive and negative controls. Indeed, when the results were analyzed using a *t*-test statistical evaluation, the EG-ethanolic extract was significantly different for both the positive and negative controls (*p* < 0.001).

Regarding results, they suggested that the antibacterial activity of EG-ethanolic decreased over time, demonstrating that methanolic solvent extraction had a better antibacterial effect than ethanol extraction. Likewise, the results showed a significant difference between the EG-methanolic and antibiotic activities. As put forward in other studies, the difference observed in the extract’s antimicrobial activity is related to the bioactive compounds’ variable chemical constituents and volatile nature [35]. These antibacterial results confirmed the previously reported EG-methanolic and EG-ethanolic extracts’ antibacterial activity, suggesting this plant is a potential resource for antibiotic discovery in the pharmacological field.

### 2.7. In Vitro Antioxidant Activity

#### 2.7.1. Ferric Reducing Power Assay (FRP)

To measure the extract reductive ability, the Fe^3+^ to Fe^2+^ transformations in the presence of the plant extract with the complex of potassium ferricyanide, trichloroacetic acid, and ferric chloride were investigated. The yellow color changed to green or blue depending on the antioxidant power presented in the experiment results, as described by Bhalodia et al. [36]. The absorbance value of the sample at 0.25% (*w*/*v*) was used to analyze antioxidant activity. The percentage of reducing power (%RP) was calculated. The results are presented in Table 5.

**Figure 6:**
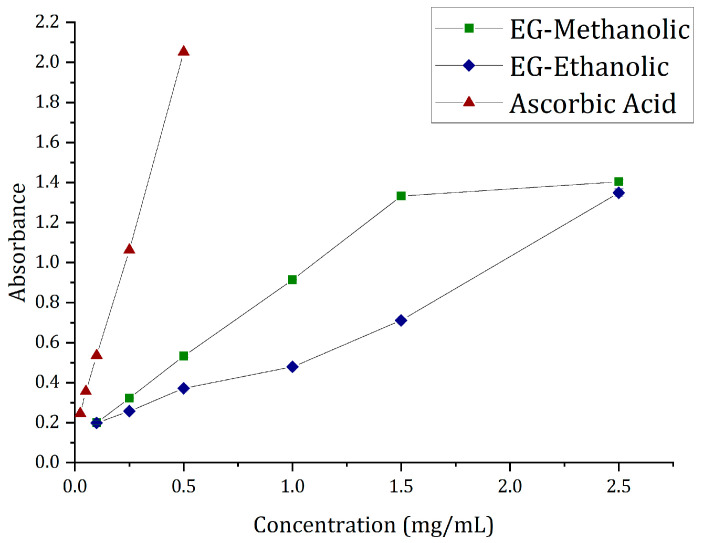
Ferric reducing power results comparison of plant extract and standard.

Evidence illustrates that the EG-methanolic extract had a remarkable potency to donate electrons to the reactive free radicals; this causes the non-reactive species to become more stable, culminating in the chain reaction characterized by free radicals [37]. The %RP of EG-methanolic is higher than EG-ethanolic extract. However, there was no statistically significant difference between the reducing power of both extracts. Correspondingly, the reducing power in the FRP assay is dependent on the plant’s chemical components due to the previous characterization. Nonetheless, excellent antioxidant activity was recorded (see Figure 6). Thus, the antioxidant ability of the extracts in inhibiting the oxidative effects of reactive oxygen species found in the reaction mixture was tested through the colorimetric method.

#### 2.7.2. Total Antioxidant Activity (TAC) by Phosphomolybdenum Method

The results could suggest the plant extracts’ antioxidant capacity, highlighting that the EG-methanolic sample has the highest TAC value of both extracts. The compounds of the extracts that may be involved in the TAC are flavonoids, carotenoids, and cinnamic acid derivatives [38,39]. The *t*-test was used to analyze the difference between the two extracts with a confidence level of 95%. Overall, the EG-methanolic extract (99.76%) showed the highest total antioxidant ability compared to the EG-ethanolic extract (51.24%; see Table 6). However, no statistical difference was found between the two extracts. Otherwise, the methanolic extractions were found to contain more antioxidant agents compared to ethanolic extractions. Although EG-methanolic and EG-ethanolic extracts were obtained from the same plant and showed the same classes of phytochemicals, the quantitative difference could result from the heterogeneous concentration of the phytochemicals. The overall antioxidant findings warrant further studies on the different isolation techniques and characterization of the secondary metabolites responsible for antioxidant activity.

### 2.8. In Vitro Anti-Hemolytic Activity

**Figure 7:**
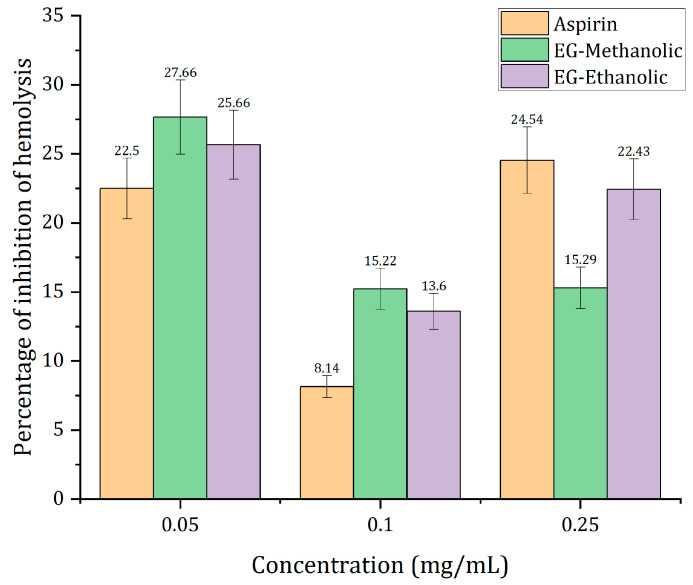
Anti-hemolytic activity of the plant extract comparing its effectiveness with the commercial aspirin.

The analysis for EG-methanolic and EG-ethanolic was applied using the formula for percentage inhibition of hemolysis; the following results have been analyzed. As shown in Figure 7, the aspirin, EG-methanolic, and EG-ethanolic extracts demonstrated anti-hemolytic activity. The inhibition of hemolysis induced by hypotonicity at different concentrations of EG-methanolic and EG-ethanolic from 8% to 30%. Aspirin was selected as a positive control as it is a well-known non-steroidal anti-hemolytic agent [40]. The percentage inhibition of hemolysis in human red blood cell stabilization by aspirin was obtained according to similar assays by Hossain et al. [41]. Specifically, EG-methanolic, the plant’s methanolic extract at 0.05 mg/mL, represented the highest value of inhibition of hemolysis, while at higher concentrations, possibly the synergism of various metabolites decreases, as working with complete extracts may result in all at high concentrations.

The results demonstrated an increasing antioxidant and anti-hemolytic activity of the EG-methanolic plant extract. The plant extract’s anti-hemolytic effect reduced the hemolysis magnitude when red blood cells were exposed to the hemolytic effect of hypotonic solution [36]. The statistical analysis was applied using 0.05 mg/mL of aspirin as a positive control. The EG-methanolic was not significantly different from aspirin’s anti-hemolytic effect. In contrast, the EG-ethanolic extract showed a significant difference compared with the control. However, both extracts decreased the stress induced by the hypotonic buffer solution on the plasma membrane to be destroyed. The presence of biomolecules in the extracts, such as flavonoids and phenols, may be responsible for this biological activity [42]. However, the therapeutic mechanism of the extracts on erythrocytes is not precisely understood.

### 2.9. Hydrogel Synthesis and Characterization

#### 2.9.1. Fourier-Transform Infrared Spectroscopy

**Figure 8:**
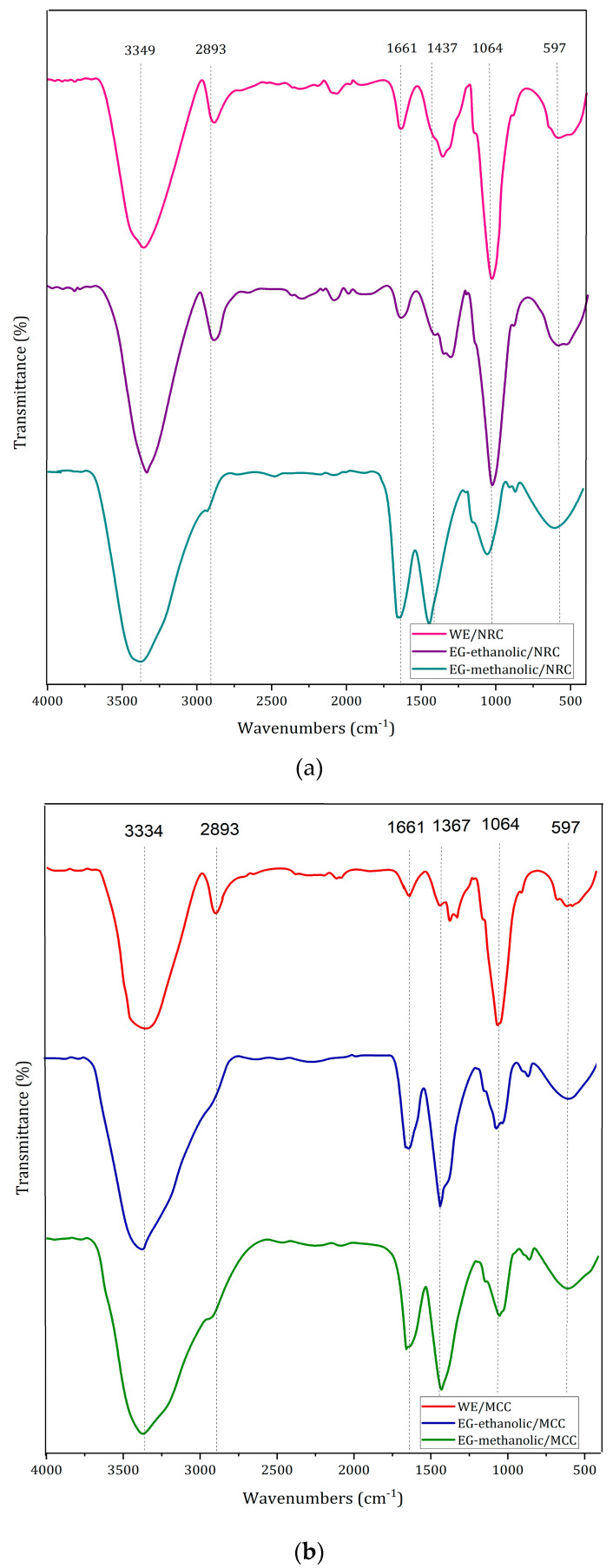
FT-IR spectrum of loaded-hydrogels compared with without extract (**a**) NRC type and (**b**) MCC type.

The hydrogels were characterized by FT-IR spectroscopy as showed in Figure 8. The 3100–3600 cm^−1^ peak corresponds to O-H stretching, while, the 2700–3000 cm^−1^ peak refers to C-H stretching. Similarly, the peak near 1700 cm^−1^ is related to C=O stretching. Finally, the peak 900–1100 cm^−1^ corresponds to the functional group C-O stretching cm^−1^. These functional groups are presented as NRC and MCC hydrogels without extract, but their difference lies in the higher intensity range of 900–1100 cm^−1^ in the NRC hydrogels [43].

The characteristic peaks of EG-methanolic are 1663.91 cm^−1^ and 1447.38 cm^−1^ for the NRC hydrogel type (a) (see Figure 8a). Meanwhile, 1661.39 cm^−1^ and 1437.28 cm^−1^ peaks are visible in EG-methanolic/MCC (see Figure 8b) hydrogels, which are not present in the xerogel spectrum. Evidence illustrates that the spectrum of hydrogels without extract differs from the extract-loaded hydrogel. The peak of 2700–3000 cm^−1^ present in the hydrogels without extract corresponds to the C-H stretching [43]. This peak is not visible in the extract-loaded hydrogels, according to Figure 3. While the peak in the region 3300–3400 cm^−1^ is related to the O-H stretching functional group in the WE/NRC and WE/MCC with solid absorption. This peak is visualized in both the hydrogels with and without extract. On the other hand, the 1600–1700 cm^−1^ corresponds to C=O stretching in the cellulose hydrogels. The % transmittance of this peak increases when the hydrogels with the extract. Finally, 1000–1100 cm^−1^ wavelength corresponds to the C-O stretching peak, whose transmittance decreases in extract-loaded hydrogels.

**Figure 9:**
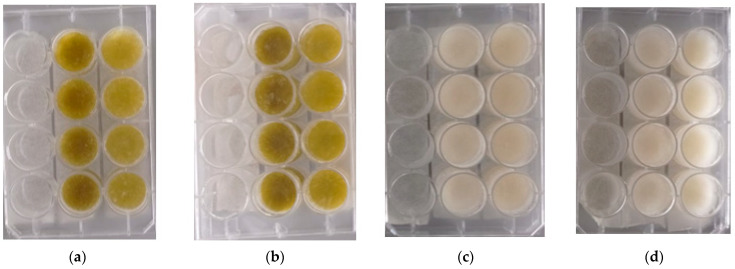
Cellulose hydrogel after encapsulation of *Eupatorium glutinosum* Lam. Extract (**a**) EG-methanolic and (**b**) EG-ethanolic, while (**c**) corresponds to before encapsulation, WE/NRC, and (**d**) to WE/MCC.

FTIR could indicate an optimal encapsulation of the extracts in the polymeric matrix of the hydrogel (see Figure 9). It was evident that the encapsulation of the functional groups of the extracts differed from the cellulose hydrogels without extract. In addition, the Fourier-transform infrared characterization between the NRC and MCC cellulose types differed only in the peak % transmittance of the previously analyzed functional groups.

#### 2.9.2. Surface Morphology of Hydrogel

**Figure 10:**
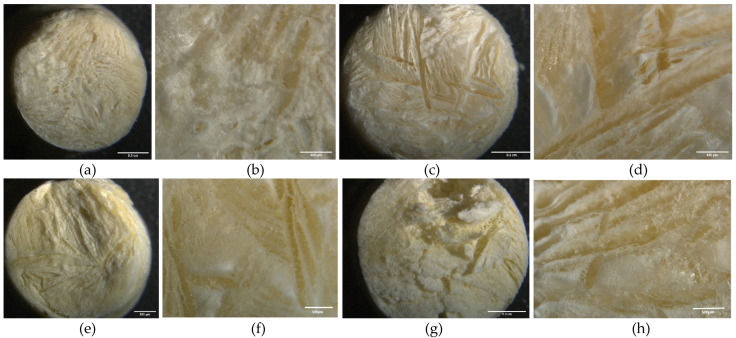
Surface morphology of plant extract-loaded hydrogel through a stereoscope with (**a**) EG-methanolic/NRC (M = 1.25), (**b**) EG-methanolic/NRC (M = 4), (**c**) EG-methanolic/MCC (M = 1.25), (**d**) EG-methanolic/MCC (M = 4), (**e**) EG-ethanolic/NRC (M = 1.25), (**f**) EG-ethanolic/NRC (M = 4), (**g**) EG-ethanolic/MCC (M = 1.25), (**h**) EG-ethanolic/MCC (M = 4).

In Figure 10, the yellowish surface color may suggest, at first glance, the encapsulation of the extracts in the NRC and MCC hydrogels. Through the stereoscope, it is visualized on the non-uniform surface. Indeed, samples showed a consistent structure. The extract particles were observed at a magnification of 4. It could be related to the solubility or the sample chemical structure of the extract and the hydrogel compounds. Hence, the white color is characteristic of microcrystalline and cotton cellulose hydrogels. The difference in color between the hydrogel without the extracts, when compared to EG-methanolic/NRC, EG-methanolic/MCC, EG-ethanolic/NRC, and EG-ethanolic/MCC, indicated encapsulation of the extract inside the polymeric matrix [44]. Unique features such as the surface topology of the loaded hydrogels developed in this research may limit their biomedical applications [45]. The physical integrity of the extract-loaded hydrogels could also be related to their mechanical strength, functionality, and adaptability [46].

#### 2.9.3. Density and Swelling of Hydrogel

The results of the hydrogel density are based on a water density of 0.997 g/cm^3^ at 4 °C [47]. The literature reports values of 1.028 g/cm^3^ for the density of microcrystalline cellulose, and the cotton cellulose density corresponds to 1.44 g/cm^3^ [48,49]. For the experimental analysis, the density of the hydrogels, including the extracts with a concentration of 0.25% (*w*/*v*) is higher than 0.1 (*w*/*v*) in both hydrogel types. Evidence illustrates that by increasing the concentration of the extracts, the number of molecules stored in the three-dimensional matrix of the hydrogel also increased. Results demonstrated that the density of the loaded hydrogel of MCC is higher than NRC. Moreover, the density of the hydrogels without the extracts (WE) is relatively higher, being 1.054 g/cm^3^ for NRC and 1.208 g/cm^3^ for MCC. The density of a hydrogel is related to its swelling ratio and other mechanical properties. Density is an influential factor in the dimensional change and the release patterns of extract from these carriers [50].

**Figure 11:**
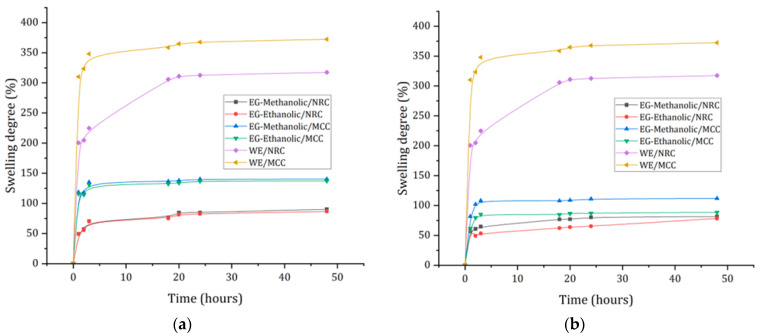
Degree of swelling of NRC and MCC hydrogel types in PBS solution with (**a**) hydrogel at relative 0.1% (*w*/*v*) and (**b**) hydrogel at relative 0.25% (*w*/*v*).

The degree of diffusion of the extract components through the hydrogel is relevant for biomedical applications with extract delivery systems [51]. The influence of the extract concentration on the swelling ratio of cellulose hydrogel in PBS at pH 7.4 is shown in Figure 11. The WE/NRC and WE/MCC exhibited a high equilibrium swelling ratio, indicating the hydrogels were superabsorbent without extract hydrogels. The amount of water stored by the hydrogels depends on the pH of the medium, the ionic strength characterized by the ionic groups, and the level of crosslinking of the polymer network [52]. The results showed significant differences between WE/NRC and WE/MCC hydrogels and loaded hydrogels containing the extracts at different concentrations. Although the chemical components of the EG-methanolic/NRC and EG-ethanolic/NRC extracts are similar, the swelling degree was different at 1 mg/mL. The same statistical analysis was conducted when EG-methanolic/NRC and EG-ethanolic/NRC were compared at 0.25 (*w*/*v*). It indicated swelling degree is affected by the solvent in the plant extraction process. In addition, the percentage of swelling degree decreased when the concentration of the EG-methanolic and EG-ethanolic extracts was increased. It may be due to the increase in bioactive compounds in the three-dimensional matrix of the hydrogel. The same statistical analysis was used to compare the EG-methanolic/MCC and EG-ethanolic/MCC, which had similar statistical results for loaded hydrogel in the two concentrations. Furthermore, it was observed that swelling in loaded hydrogels corresponded to the 25−85% range, while the hydrogel without extract in the NRC and MCC hydrogel showed a swelling degree of 300%.

Results showed optimal release of extracts under the conditions of a dynamic pH-based study with phosphate buffer solutions (pH 7.4). The microcrystalline cellulose demonstrated a higher degree of swelling than NRC cellulose hydrogels. The WE/MCC swelled up to 375%. Moreover, the extract-loaded MCC hydrogel showed a higher swelling value than the NRC samples. The swelling values of the hydrogel increased considerably in the first 5 h, followed by augmenting steeply up to 24 h. Thus, the degree of swelling is inversely proportional to the concentration of the extract [53] for EG-methanolic and EG-ethanolic. Hydrogels with methanol extracts showed better swelling than ethanolic extracts. Finally, the swelling process depends on the extract concentration because it could affect the rate and speed of release.

#### 2.9.4. Loaded-Hydrogel Release Profiles

**Figure 12:**
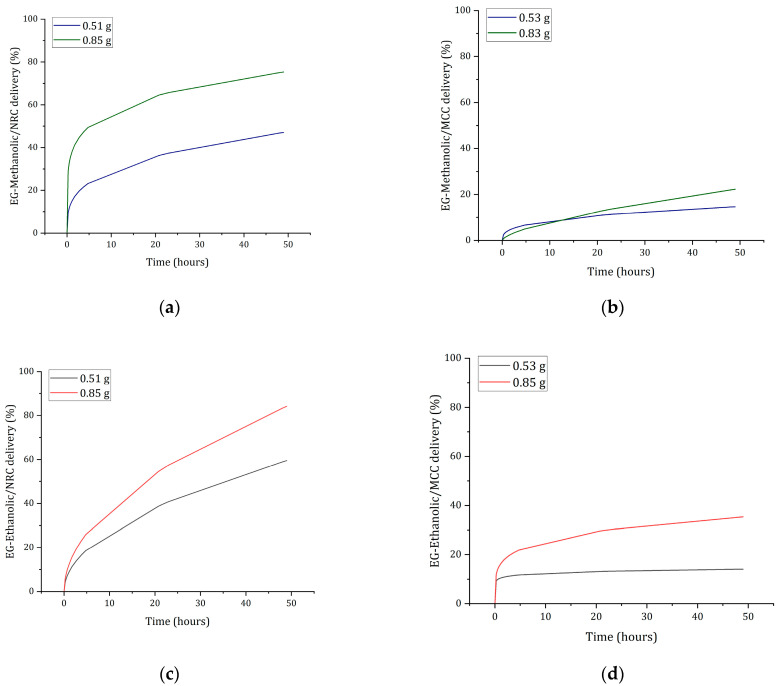
Plant extracts releasing data from the NRC and MCC-loaded hydrogel in PBS pH (7.4) with (**a**) EG-methanolic/NRC, (**b**) EG-methanolic/MCC, (**c**) EG-ethanolic/NRC, and (**d**) EG-ethanolic/MCC hydrogel delivery profiles.

The experimental result of the in vitro extract released from the cotton cellulose and crystalline cellulose in PBS at a pH of 7.4 is shown in Figure 12. The release studies were conducted by keeping two different hydrogel weights. After fitting experimental data to the Korsmeyer-Peppas model, it was determined that the increased extract amount released is proportional to the hydrogel weight. However, a considerable difference between the release achieved by cotton cellulose and microcrystalline cellulose could be influenced by the degree of crosslinking of the polymers and their physical properties [54]. The extracts release occurred owing to the pore size increase in the matrix network due to swelling loaded hydrogel beads [55] affecting each extract release. Extract release profiles differ between NRC and MCC-loaded hydrogel types.

Figure 12 indicates that EG-methanolic/NRC and EG-ethanolic/NRC demonstrated an extract releasing up to 80%. Meanwhile, extract release in EG-methanolic/MCC and EG-ethanolic/MCC only achieved less than 30% in both hydrogel types. Moreover, the EG-methanolic/NRC and EG-ethanolic/NRC samples are significantly different in other terms, demonstrating that the release profiles are independent of the amount of hydrogel used. In contrast, the EG-methanolic/MCC released profile was not different scientifically at 0.5175 g and 0.8500 g. The release of the EG-methanolic extract showed the same trend in percent release. Nevertheless, EG-ethanolic/MCC did not show a significant difference at 95%.

The release profile can be influenced by the extract’s solubility and the hydrogel’s size or shape, which directly influence the data’s model fit of the data [4]. Through the results of the release profiles, the dissociation of the trapped particles through the mechanism of diffusion through the hydrogel was verified.

#### 2.9.5. Biological Activity of Loaded-Hydrogel

**Figure 13:**
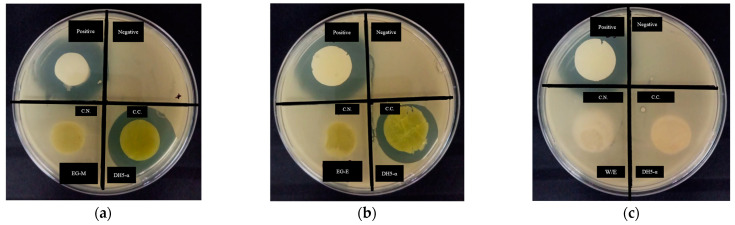
Antibacterial activity of the extract-loaded hydrogels with (**a**) EG-methanolic/NRC and EG-methanolic/MCC, (**b**) EG-ethanolic/NRC and EG-ethanolic, and (**c**) WE/NRC and WE/MCC.

The inhibition halo reported the antibacterial activity of cellulose hydrogel. The positive control was kanamycin, a commercial antibiotic at 5 mg/mL, while the extract concentration of hydrogel was 0.25% (*w*/*v*) for the EG-methanolic and EG-ethanolic samples. Figure 13 shows the inhibition halo of extract-loaded hydrogel after 24 h of incubation. Then, halo forming on the microcrystalline cellulose hydrogels demonstrated the antibacterial activity of the hydrogels loaded with the plant extract against *E.coli* DH5-α growth [55].

Therefore, the loaded hydrogel with the extract and kanamycin delivered from the microcrystalline cellulose hydrogels showed antibacterial effect against *E. coli* DH5-α growth. The difference in antibacterial activity detected in the MCC-type hydrogels compared to the NRC type may be related to the amount of extract released by the hydrogel and, therefore, to the swelling rate ratio. The EG-methanolic/MCC hydrogel produced an inhibition halo of 3.17 cm for DH5-α. Meanwhile, the EG-ethanolic/MCC formed an inhibition halo of 2.93 cm. In particular, the positive control (kanamycin) showed an inhibition halo of 3.43 cm for EG-methanolic/MCC and 3.49 cm for EG-ethanolic/MCC. Through a *t*-test, it was determined that the EG-methanolic/MCC and EG-ethanolic/MCC are not significantly different, i.e., there is no difference in the antibacterial activity of the extract-loaded hydrogel. Similarly, compared to the biological activity recorded by the extract-loaded hydrogel of type MCC compared to its positive control, at a significance level of 95%, it is determined that they are not significantly different.

These results are consistent with the well-known antibacterial activity of kanamycin [56]. In contrast, the WE/NRC and WE/MCC-loaded hydrogels had no antibacterial activity, as was expected. Moreover, average bacterial growth was observed in the negative control section (see Table 7).

The ability of EG-methanolic/MCC and EG-ethanolic/MCC hydrogels to inhibit the growth of bacteria could be due to the specific presence of a specific secondary metabolite or synergy [57]. The diverse chemical groups interact with bacterial membranes due to hydrophobic interactions, thus enhancing cell membrane outgrowth and harming the structure of bacterial cells [30]. Finally, the positive antibacterial result of extract-loaded hydrogel could be useful for cellulose-based biomedical devices.

## 3. Materials and Methods

### 3.1. Materials

*Eupatorium glutinosum* Lam. leaves were purchased from the Ibarra herbal market, Imbabura, Ecuador. The reagents used were methanol (Merck, Darmstadt, Germany), ethanol (Merck, Germany), sodium chloride (Merck, Germany), dimethyl sulfoxide (Sigma-Aldrich, St. Louis, MO, USA), LB Broth Miller (Difco Laboratories, Detroit, MI, USA), Sigma Cellulose Type 101 Cellulose (Sigma-Aldrich, USA); microcrystalline cellulose (Thermo Scientific™, USA), potassium chloride (Sigma-Aldrich, USA), disodium hydrogen phosphate anhydrous (Scharlau, Barcelona, Spain), and potassium phosphate monobasic (Thermo Scientific™, USA).

### 3.2. Extracts Preparation of Medicinal Plant

*Eupatorium glutinosum* Lam. (EG) leaves were sun-dried for one day, and an oven-drying chamber was used for 24 h. Then, it was pulverized in the shredding machine until it obtained 1–5 mm particles. Then, 100 mL of solvent was added, and the mixture was macerated for 15 days at room temperature. The mixture was filtered. Subsequently, the filtrates were rotoevaporated until the concentrate of the plant extract was obtained. The samples were stored at 4 °C, protected from sunlight, until used for chemical analysis. The extract yield was calculated with the method described by Dhanani et al. [58].

### 3.3. Phytochemical Screening

The methanolic and ethanolic extracts of phytochemical compounds were analyzed by several methods to standardize the reported procedure in [16,17,18] with slight modifications to identify secondary metabolites. The secondary metabolites identified corresponded to quaternary alkaloids and/or free amino acids, coumarins, lactones, saponins, terpenes, steroids, terpenoids, reducing sugar, flavonoids, quinines, phenols, tannins, glycosides, mucilage, and carbohydrates.

### 3.4. Thin Layer Chromatography

In thin-layer chromatography (TLC), precoated plates (silica gel 60 F_254_) were applied with 2–3 µL of the concentrated extract using a capillary tube. Plates were marked about 1 cm with a pencil on the bottom side and waited until ¾ parts of the solvent covered the plates. The TLC was carried out in a chromatographic tank using chloroform/acetone/formic acid (7.5:1.65:0.85) as a solvent system [59]. After drying, the TLC plate was revealed by a UV-light lamp (365 nm) and sulfuric acid/vanillin chemical developer.

### 3.5. Spectrophotometric Characterization with UV/Vis

The UV/Visible spectroscopy of the plant extracts allowed the identification of the profile by scanning samples in the visible UV/Vis region to obtain the characteristic peaks [37]. For the UV/Vis spectrum, the dried extracts were diluted in different concentrations to obtain the point on the curve where there is significant absorbance. The corresponding amount of the extract was diluted in distilled water to obtain the different concentrations of 0.01%, 0.05%, 0.025%, and 0.01% (*w*/*v*). The absorbance was measured with a quartz cell at room temperature. This analysis was conducted using the Lambda 1050 UV/Vis/NIR spectrophotometer (PerkinElmer, Waltham, MA, USA). The wavelength range used for chemical analysis was from 250 nm to 800 nm.

### 3.6. Fourier-Transform Infrared Spectroscopy

The Fourier-transform infrared spectroscopy (FTIR) spectrum of all extracts was obtained in the infrared region with a wavelength between 4000 and 400 cm^−1^. This method is used for functional group identification by % transmittance value in the spectrum. A small quantity of EG-methanolic and EG-ethanolic were placed in the Cary 630 Fourier-transform infrared spectrometer (Agilent Technologies, Stockport, UK) to elucidate the structure of the unknown composition and the intensity of the absorption spectra associated with molecular composition or chemical functional groups [60]. The spectrum information was registered for each extract.

### 3.7. Antibacterial Activity

#### 3.7.1. Agar-Diffusion Essay

The previously prepared plant extracts were then evaluated for their potential antibacterial activity against three strains of Gram-negative *Escherichia coli* species, more exactly, TG1 (a derivative strain of *E. coli* JM101, which has neither modification nor restriction on transformed exogenous DNA) [61], DH5-α (a typical engineered *E. coli* widely used in the laboratory) [62], and E410A5 (corresponding to *E. coli* construct used in molecular biology) [63]. Using Luria-Bertani broth, Miller, the TG1, DH5-α, and E410A5 strains were grown overnight at 37 °C with 160 revolutions per minute [35]. Then, EG-methanolic and EG-ethanolic extracts were dissolved in 3 mL of distilled water to evaluate the antimicrobial activity at different concentrations of 2.0%, 1.50%, 1.0%, 0.5%, 0.25%, 0.15%, 0.01%, 0.05%, 0.025%, and 0.01% (*w*/*v*). Based on the standard McFarland turbidity values, a value of 3 was obtained in the initial bacterial suspension with an approximately 1 × 10^8^ colony-forming units/mL (CFU/mL) cell density by a factor of 9 [64]. This value was confirmed by measuring OD600 equal to 0.602 absorbance measurement on NanoDrop™ 2000 Spectrophotometer (Thermo Fisher Scientific™, USA) [65]. Fresh Luria Bertani agar, Miller (LBA, Miller; Difco Laboratories, USA) medium was poured into several sterile Petri dishes, and 300 µL of each *E. coli* strain from the initial suspension was seeded in these plates. Then, different extracts at the previous concentrations were placed on the top of LBA, Miller plates. Kanamycin was used as the positive control for its well-known antibacterial capacity at 5% (*w*/*v*). The samples were incubated at 37 °C for 24 h. The MBC was determined as the lowest concentration inhibiting bacteria growth. Antimicrobial activity was detected by measuring the zone of inhibition that appeared after incubation. All assays were performed in triplicate.

#### 3.7.2. Time-Kill Curve Analysis

The time-kill kinetics test or antimicrobial efficacy testing of EG-methanolic and EG-ethanolic extracts was evaluated using DH5-α *Escherichia coli* after the results of MBC assays. For cell culture optical density measurements, the NanoDrop^TM^ 2000 spectrophotometer was used in an in vitro time-kill methodology bioassay experiment [66]. The blank was an LB broth, Miller culture medium without extract, and the wavelength measurement was 600 nm. The positive control was kanamycin at 2.0% (*w*/*v*) concentration. In the same way, the negative control was LB broth, Miller culture, plus bacteria at 1x10^8^ CFU/mL without antibiotic or extract. Meanwhile, the plant extract was prepared at the same concentration for EG-methanolic and EG-ethanolic samples and then incubated together with the same bacterial suspension. Using LBA Miller slants, 25 µL of *E. coli* DH5-α bacteria was cultivated at 37 °C with 160 revolutions per minute for 24 h [35]. The volume taken from the initial bacterial suspension was calculated according to Beat et al. [67], with slight modifications to the concentration-optical density correlation during the first 5 h of growth culture.

### 3.8. In Vitro Antioxidant Activity

#### 3.8.1. Ferric Reducing Power Assay (FRP)

Ferric reducing antioxidant power was performed to determine Fe^3+^ to Fe^2+^ by measuring the absorbance of the sample and standard in 700 nm by the UV/Vis spectrophotometer because it is the absorption maximum to the ferric, ferrous complex [68]. The antioxidant compound in the extracts was recognized by the antioxidant assay activity recorded for forming complexes with the metal atoms present, specifically copper and iron [69]. Three replicates of each sample were prepared for the assay. A blank was prepared without adding the extract to the antioxidant complex. The standard reagent was ascorbic acid at different concentrations for the assays.

The calibration curve equation for FRP analysis based on the resulting absorbance versus concentration was obtained by y = mx + b, where m is the slope and b is the y-intercept. The reduction phenomena were expressed in μg ascorbic acid equivalents/g based on the ascorbic acid equivalent antioxidant activity and μgAA micrograms of ascorbic acid (AEAC). Therefore, the percentage of reducing power (%RP) was calculated using the guidelines of Xiao et al. [70].

#### 3.8.2. Total Antioxidant Activity (TAC) by Phosphomolybdenum Method

The chemical principle to assess the total antioxidant capacity (TAC) is based on the plant extract possessing antioxidant compounds through the reduction of Mo (VI) to Mo (V) [71]. The chemical complex allowed us to measure the total antioxidant activity by the phosphomolybdenum method. The extracts were evaluated according to the method described in [38] for antioxidant capacity. Samples and standards were prepared at the same concentration as the previous FRP assay. This is shown by the green coloration change indicating phosphomolybdenum complex V, whose absorbance was measured at 695 nm using a UV/VIS/NIR Lambda 1050 spectrophotometer (PerkinElmer, Waltham, MA, USA) [39]. The total antioxidant activity is expressed in grams equivalent of ascorbic acid, as well as the calibration curve, with ascorbic acid [72]. The total antioxidant activity was calculated for the extracts, and the standard’s highest concentration

### 3.9. In Vitro Anti-Hemolytic Activity

The required concentrations of plant extracts and aspirin (positive control) were prepared. Human red blood cells (HRBC) have been used for anti-hemolytic in vitro studies [73]. Blood was obtained from a volunteer with the restriction of not consuming any anti-hemolytic drug fourteen days before the test. Therefore, a 5 mL commercial tube with EDTA-K3 VanTubo and a sterile needle were employed; and 15 mL (3 dips) of human blood was obtained and slowly mixed with the coagulant. The Committee on Ethics and Research on Humans of Solca Hospital approved the experiment (Quito, Ecuador). Therefore, it was centrifuged at 3000 rpm for 5 min, and the supernatant was eliminated. The cell suspension was washed with saline solution (0.9% *w/v* NaCl) and centrifuged at 3000 rpm for 5 min, threefold. Finally, the last resuspension was prepared with 40% (*v/v*) of phosphate-buffered saline (PBS) solution at pH 7.4.

The procedure corresponds to hemolysis induced by hypotonicity results. For this procedure, 2 mL of 0.9% saline solution, 1 mL of PBS solution, and 1 mL of EG-methanolic and EG-ethanolic extract were prepared in a falcon tube. Subsequently, 500 μL of the previous blood preparation was added to the falcon tube. Then, samples were incubated for 30 min at 37 °C, and tubes were centrifuged for 20 min at 3000 revolutions per minute. The supernatant was collected, and the absorbance of each extract solution was measured at 560 nm as an indicator of the degree of hemolysis [74]. The percentage of hemolysis inhibition was calculated according to Aidoo et al. [75] as follows:

$$Inhibitionofhemolysis=\left[\frac{OD_{w}-OD_{E}}{OD_{w}}\right]*100$$

where $OD_{w}$ is the optical density of the hypotonic buffer without extract, and $OD_{E}$ is the optical density of the hypotonic buffer with the extract.

### 3.10. Synthesis and Characterization of Hydrogel

#### 3.10.1. Cellulose Hydrogel Synthesis

This section aims to create cellulose hydrogels with the EG-methanolic and EG-ethanolic extracts to study their use in biomedical applications. Sigma Cellulose Type 101/cotton cellulose (NRC) and Microcrystalline Cellulose (MCC) were used to analyze the difference between chemical and physical cellulose structures with and without encapsulation of extract.

#### 3.10.2. Fourier-Transform Infrared Spectroscopy of Loaded Hydrogel

The Fourier-transform infrared (FTIR) spectra were recorded using a Cary 630 Fourier Agilent spectrometer in the 4000–450 cm^−1^ frequency range, at a resolution of 4 cm^−1^ [76]. FTIR spectroscopy of the lyophilized EG-methanolic/NRC, EG-methanolic/MCC, EG-ethanolic/NRC, and EG-ethanolic/MCC hydrogels was carried out and compared with WE/NRC and WE/MCC samples, where WE means without extract hydrogel.

#### 3.10.3. Surface Morphology of Hydrogel

The M205-C stereo microscope (Leica Microsystems, Heerbrugg, Switzerland) was used to analyze the morphology of the hydrogels and observe their surfaces in detail [77]. A magnification of 1.5 and 4 was used on EG-methanolic/NRC, EG-methanolic/MCC, EG-ethanolic/NRC, EG-ethanolic/MCC, WE/NRC, and WE/MCC for surface analysis. The three-dimensional structure of the cellulose hydrogel suffered swelling in the presence of an aqueous medium, and the ability to retain different volumes of water was evaluated.

#### 3.10.4. Density and Swelling of Hydrogel

Cellulose hydrogel density was determined by Zu et al. [78] based on the mass per unit volume, then hydrogel samples were weighed, and dimensions were obtained. Correspondingly, the swelling degree of the hydrogel was studied in order to analyze the maximum level of liquid that can be absorbed depending on the degree of swelling [53]. For this purpose, a PBS solution adjusted to a pH of 7.4 at 37 °C was used. The measure started with a dried hydrogel and weight measure. Then, samples were immersed in PBS at 37 °C and weighed at 0, 3, 6, 9, 12, 24, and 28 h. The objective of this procedure was to study the absorption capacity. The degree of swelling was then calculated, as previously reported by Gull et al. [79].

#### 3.10.5. Loaded-Hydrogel Release Profiles

Extract-loaded hydrogels EG-methanolic/NRC, EG-methanolic/MCC, EG-ethanolic/NRC, and EG-ethanolic/MCC, as well as WE/NRC and WE/MCC, were dried at 45 °C. Then cellulose hydrogels were immersed in 4 mL of PBS at pH 7.4. The experiment was carried out in the MaxQ™ 4450 Benchtop Orbital Shaker (Thermo Scientific™, USA) at 25 revolutions per minute for 72 h at 37 °C. The samples were realized in triplicate, and the weight variation of the hydrogels, with approximate values of 500 mg and 850 mg, was evaluated. NanoDrop™ 2000 spectrophotometers measured the amount of extract released through periodic measurements each hour. Based on the UV/Vis profile of the extracts, the maximum peak was recorded for EG-methanolic/NRC, EG-methanolic/MCC at 295 nm, and EG-ethanolic/NRC, EG-ethanolic/MCC, at 285 nm. A 5 µL aliquot was taken from the buffer where the hydrogels were immersed. The absorbance values correlating to the release profiles were fitted to the linear regression equation corresponding to the Korsmeyer-Peppas math model based on the drug released from the cellulose hydrogel literature reference [80,81]. The specific peaks of absorbance of the extracts were obtained from the previous UV/Vis analysis.

### 3.11. Antimicrobial Activity of Loaded-Hydrogel

The biological activity of the loaded hydrogel with plant extract was studied in the *E. coli* strain DH5-α due to the results of the antibacterial activity previously obtained from this bacterial strain. For the inoculum’s preparation, 2 mL of LBA, Miller, and 25 µL of the DH5-α bacteria suspension at 1 × 10^8^ CFU/mL were mixed and then incubated at 37 °C with 160 revolutions per minute for 24 h. For the hydrogel preparation, the Kirby-Bauer (KB) method was applied in the EG-methanolic/NRC, EG-methanolic/MCC, EG-ethanolic/NRC, and EG-ethanolic/MCC-loaded hydrogels to evaluate the antimicrobial activity when compared to WE/NRC and WE/MCC-loaded hydrogels against DH5-α [82]. For loaded hydrogel testing, disc-shaped samples were prepared with a height of 0.7 cm and a diameter of 1.9 cm. The hydrogel loaded with the extract was washed until it reached a pH close to 7.0. To the LBA, Miller Petri dishes, a volume of 300 µL of the inoculum’s preparation was added, and the loaded hydrogels were placed over the medium. After 24 h of incubation at 37 °C, the inhibition halo was measured for each loaded hydrogel.

### 3.12. Statistical Analysis

The data generated from quantitative assays were subjected to *t*-test statistical analyses using R software (version 4.2.2; http://www.R-project.org). The difference was considered statistically significant if *p* < 0.05. All tests were treated in three replicates.

## 4. Conclusions

It was determined that the EG-methanolic and EG-ethanolic natural extracts were successfully obtained through methanol and ethanol extractions with positive results in the in vitro studies of antibacterial, antioxidant, and anti-hemolytic activities. In addition, the components of the extracts were encapsulated in two types of cellulose hydrogels. The characterization results, swelling, release profile, and biological activity suggest their potential application as a controlled release system of natural compounds. The outcomes provide insight that the synthesis and characterization of the two extracts (methanolic and ethanolic) from *Eupatorium glutinosum* Lam. could suggest the possible bioactive interest, making the used species plants good candidates for drug development.

The biological activity of plant extracts may suggest promising medical uses for pharmacological applications due to their ability to break the membrane of cell lines, be a source of natural antioxidants, and prevent the breakdown of the erythrocyte membrane.

The hydrogel formulation allowed the encapsulation conditions of the bioactive compounds of plant extracts to be tested through the characterization of the loaded hydrogels as well as the swelling, release, and antibacterial activity assays and their potential biomedical applications. There was an effective enclosure of natural extracts in cotton and microcrystalline cellulose hydrogels.

## Figures and Tables

**Table 1:** Ethanolic and methanolic yield of extracts.

Extracts	Dry Plant (g)	Solvent (mL)	Dry Extract (g)	Yield (%)
EG-methanolic	15.51	150	1.39	8.97
EG-ethanolic	14.98	150	0.95	6.37

**Table 2:** Phytochemical screening of methanolic and ethanolic extracts.

Secondary Metabolite	Performed Test	EG-Methanolic	EG-Ethanolic
Quaternary alkaloids and/or free amino acids	Mayer test	++	+
	Dragendorff test	+++	++
	Wagner test	++	+
Coumarins and lactones	NaOH test	+	+
Saponins	Frothing test	+++	+++
Terpenes and steroids	Liebermann-Buchard test	+	++
Terpenoids	Salkowski test	+++	+++
Flavonoids	Alkaline reagent test	+	+
	Shinoda test	+	+
Quinones	Bontrager test	−	+++
	Acid-base test	−	+
Phenols and tannins	Ferric chloride test	+++	++
Glycosides	Keller-Killiani test	−	++
Carbohydrates	Benedict’s test	+	+

Legend: (−) means absent, (+) means present, (++) means moderate present, (+++) means high present.

**Table 3:** UV/Vis profiles of secondary metabolites.

Wavelength (nm)	EG-Methanolic (nm)	EG-Ethanolic (nm)	Metabolites	Ref
234–676	295 320 410 615 675	285 325 405 615 675	Alkaloids, flavonoids, and phenol	[25]
230–350	295 320	285 325	Flavonoids and their derivates	
350–500	410	405	Tannins, carotenoids, and carotenoids	[26]
600–700	675	615 675	Chlorophyll	[27]

**Table 4:** Antimicrobial screening test of the effective extract against *E. coli* DH5-α.

	Inhibition Halo (mm)						Positive Control	Negative Control
Plant Extract mg/mL	1.5	2.5	5	10	15	20	Kanamycin (50 mg/mL)	Water Type 1
EG-methanolic	-	1.9	2.6	2.8	2.3	2.5	13.0	-
EG-ethanolic	-	0.9	2.1	2.3	2.0	1.8	15.0	-

**Table 5:** FRAP value in AEAC (mg AA/g) and reducing power (%RP).

Plant Extract	FRAP Value in AEAC (mg AA/g)	Reducing Power (%RP)
EG-methanolic	7.96	68.41
EG-ethanolic	7.60	65.68

**Table 6:** Total antioxidant activity of plant extracts.

Plant Extract	Total Antioxidant Activity (%)
EG-methanolic	99.76
EG-ethanolic	51.24

**Table 7:** Antibacterial activity of extract-loaded hydrogel against *E. coli* DH5-α growth.

Plant Extract		Diameter (cm)	
	/NRC	/MCC	Positive Control
EG-methanolic	NA	3.17	3.43
EG-ethanolic	NA	2.93	3.49
Without extract	NA	NA	3.52

Legend: NA = non-active.

## Data Availability

All data is available on the manuscript or by requesting to the corresponding authors.

## Sample Availability

Samples of the compounds are available from the authors.
